# Alcohol Consumption and Breast and Ovarian Cancer Development: Molecular Pathways and Mechanisms

**DOI:** 10.3390/cimb46120866

**Published:** 2024-12-20

**Authors:** Francesca Fanfarillo, Brunella Caronti, Marco Lucarelli, Silvia Francati, Luigi Tarani, Mauro Ceccanti, Maria Grazia Piccioni, Loredana Verdone, Micaela Caserta, Sabrina Venditti, Giampiero Ferraguti, Marco Fiore

**Affiliations:** 1 Department of Experimental Medicine, Sapienza University of Rome, 00161 Rome, Italymarco.lucarelli@uniroma1.it (M.L.);; 2 Department of Maternal Infantile and Urological Sciences, Sapienza University of Rome, 00161 Rome, Italy; 3 SITAC, Società Italiana per il Trattamento dell’Alcolismo e le sue Complicanze, 00185 Rome, Italy; 4 Institute of Molecular Biology and Pathology (IBPM-CNR), 00161 Rome, Italy; 5 Department of Biology and Biotechnologies “Charles Darwin”, Sapienza University of Rome, 00161 Rome, Italy; 6 Institute of Biochemistry and Cell Biology (IBBC-CNR), Department of Sensory Organs, Sapienza University of Rome, 00161 Rome, Italy

**Keywords:** alcohol, cancer, breast, ovaries, DNA, oxidative stress, inflammation

## Abstract

Alcohol consumption has been consistently linked to an increased risk of several cancers, including breast and ovarian cancer. Despite substantial evidence supporting this association, the precise mechanisms underlying alcohol’s contribution to cancer pathogenesis remain incompletely understood. This narrative review focuses on the key current literature on the biological pathways through which alcohol may influence the development of breast and ovarian cancer. Key mechanisms discussed include the modulation of estrogen levels, the generation of reactive oxygen species, the production of acetaldehyde, the promotion of chronic inflammation, and the induction of epigenetic changes. Alcohol’s impact on estrogenic signaling, particularly in the regulation of estrogen and progesterone, is explored in the context of hormone-dependent cancers. Additionally, the role of alcohol-induced DNA damage, mutagenesis, and immune system modulation in tumor initiation and progression is examined. Overall, this review emphasizes the importance of alcohol as a modifiable risk factor for breast and ovarian cancer and highlights the need for further research to clarify its role in cancer biology.

## 1. Introduction

Alcohol consumption has been linked to several types of cancers in humans [1,2,3,4]. Alcohol consumption is prevalent worldwide, with varying patterns of use across different regions and cultures. The data on global alcohol consumption in 2019 shows that an estimated 400 million people aged 15 years and older live with alcohol use disorders, and an estimated 209 million live with alcohol dependence [5]. Alcohol abuse—defined as the harmful or hazardous use of alcohol leading to adverse health effects—is a significant public health concern [6,7,8].

The global burden of alcohol-related harm is estimated to cause 5.3% of all deaths and 5.1% of the global disease burden, according to the Global Burden of Disease Study [9]. Alcohol abuse is typically characterized by excessive consumption, binge drinking, and alcohol dependence, which have a direct impact on morbidity and mortality [10,11]. Long-term alcohol abuse increases the risk for a range of diseases, including liver cirrhosis, cardiovascular disease, neurological disorders, and several types of cancer, particularly those of the gastrointestinal tract, liver, and breast [12,13,14]. Epidemiological studies have demonstrated a clear association between alcohol abuse and the increased incidence of certain malignancies, making it a major modifiable risk factor for cancer [15,16,17]. There is evidence that alcohol consumption promotes the onset of tumors in many organs, for example, the liver, pancreas, larynx, oral cavity, and esophagus [18,19].

Numerous studies have highlighted an increased risk of colorectal cancer [20,21,22]. Additionally, alcohol consumption is a well-known risk factor for breast cancer in women [23,24,25,26]. Breast cancer is a multifactorial disease whose etiology is associated with multiple risk factors related to diet, lifestyle, and hormones [27,28,29,30]. Among these factors, alcohol consumption has been linked to a higher risk of breast cancer [31,32].

The link between alcohol consumption and ovarian cancer has been less thoroughly explored, but emerging evidence suggests a potential correlation [33,34]. The proposed mechanisms may involve hormonal modulation and the effect of alcohol on inflammation and cellular proliferation within ovarian tissue [35]. Alcohol consumption is a well-established risk factor for breast cancer, primarily through its effects on estrogen metabolism, oxidative stress, and inflammation. Alcohol promotes the development of estrogen receptor-positive (ER+) breast cancer by elevating estrogen levels and causing DNA damage [36]. In ovarian cancer, the relationship is less clear but may involve similar mechanisms of hormonal modulation, oxidative stress, and inflammation. Although the role of estrogen in ovarian cancer is less well-defined, alcohol’s impact on estrogen levels and its potential to increase oxidative damage and inflammation are key factors in its association with ovarian cancer risk [37].

## 2. Materials and Methods

Between June and October 2024, for this narrative review, a wide literature exploration was carried out to disclose relevant articles (PubMed, Scopus, and WOS). The papers were chosen with the following keywords: “Alcohol”, “Ethanol”, “DNA”, “Oxidative Stress”, “Cancer”, “Breast” and “Ovaries”. We did not filter the year of publication. The chosen papers were meticulously reviewed and assessed by all authors to disclose studies that potentially encounter the aim of this narrative review.

Key inclusion criteria were (1) original studies that dealt with alcohol, ethanol, DNA, oxidative stress, cancer, breast, and ovaries and (2) articles in English. Editorials, case reports, and letters were excluded from this paper. Any divergences between the authors of this study were resolved through a consensus approach.

## 3. Molecular Mechanisms of Alcohol-Mediated Carcinogenesis

**Figure 1:**
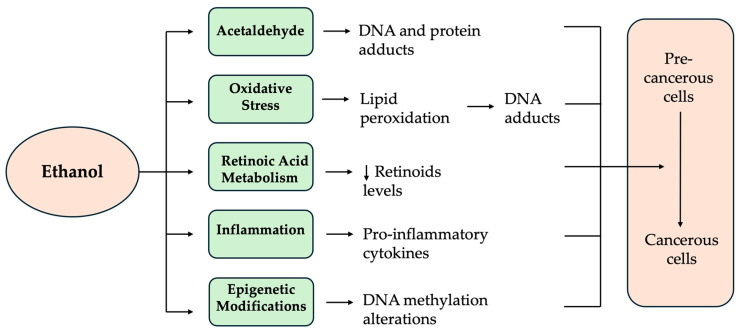
Mechanisms of alcohol-mediated carcinogenesis. Alcohol metabolism produces acetaldehyde, which can bind to DNA and proteins, forming DNA adducts. Another mechanism involves the induction of oxidative stress and the generation of ROS, which can lead to lipid peroxidation and the production of aldehydes capable of binding to DNA and creating etheno-DNA-protein adducts. Furthermore, alcohol can disrupt retinoid metabolism by inhibiting the conversion of vitamin A to retinoic acid. Chronic alcohol consumption can also promote the recruitment of monocytes and macrophages into the tumor microenvironment, producing pro-inflammatory cytokines. Lastly, chronic alcohol consumption can affect the individual’s epigenetic profile by depleting intracellular methyl group reserves that support the epigenome. The short down pointing arrow indicates decrease.

Alcohol-mediated carcinogenesis involves multiple molecular mechanisms that contribute to cancer development [38]. When ethanol is metabolized, it is converted primarily to acetaldehyde by the enzyme alcohol dehydrogenase (ADH), a potent carcinogen that can form DNA adducts, leading to mutations. The accumulation of reactive oxygen species (ROSs) during alcohol metabolism causes oxidative stress, which can damage cellular components, including DNA, proteins, and lipids, further contributing to genetic mutations and genomic instability [39]. Alcohol also influences hormonal pathways, especially in hormone-sensitive cancers like breast cancer, by increasing levels of estrogen through the upregulation of aromatase activity, promoting cell proliferation [40]. Additionally, alcohol-induced inflammation activates pathways like NF-kB, which can drive tumorigenesis by promoting cell survival, proliferation, and metastasis [41]. These combined molecular effects, including DNA damage, oxidative stress, hormonal modulation, and inflammation, all contribute to the carcinogenic potential of alcohol in various tissues (Figure 1).

### 3.1. Oxidative Stress

One of the mechanisms by which ethanol contributes to carcinogenesis is through the induction of oxidative stress [42,43].

Oxidative stress can result from the activation of some pathways that produce reactive oxygen species (ROSs), such as hydrogen peroxide and superoxide anion. One of these pathways involves the increase in the activity of CYP2E1, which produces ROS through the oxidation of ethanol to acetaldehyde [44]. It has been shown that excessive alcohol consumption increases CYP2E1 expression in the esophagus. Other sources of ROS resulting from ethanol metabolism are represented by the mitochondrial respiratory chain and some cytosolic enzymes [45].

ROS are highly reactive species that can lead to lipid peroxidation, resulting in the production of aldehydes that can form etheno-DNA adducts through their binding with DNA [46,47]. These DNA adducts are represented by 1,N6-ethenodeoxyadenosine and 3,N4-ethenodeoxycytidine, which are highly mutagenic [48].

Moreover, ROS can lead to the activation of nuclear transcription factor (NF-kB) by acting as messengers in intracellular signaling pathways. ROS interfere with mitogen-activated protein kinase signaling pathways and upregulate vascular endothelial growth factor (VEGF) and monocyte chemoattractant protein-1 (MCP-1), thus increasing cell proliferation and metastasis [49].

### 3.2. Acetaldehyde Production

Alcohol metabolism leads to the production of acetaldehyde through the action of enzymes such as cytochrome P-450 2E1 (CYP2E1), alcohol dehydrogenase (ADH), and bacterial catalase [50]. Acetaldehyde has a carcinogenic and genotoxic action. Being highly reactive towards DNA, acetaldehyde can bind to DNA and form DNA adducts (such as N2-ethylidene-2-deoxyguanosine, N2-ethyl-2-deoxyguanosine, N2-propano-2-deoxyguanosine (PdG), and N2-etheno-2-deoxyguanosine) [51], which can lead to blocking of DNA synthesis and repair. These DNA adducts can induce double-strand breaks, DNA point mutations, sister chromatid exchanges, and structural changes in chromosomes [52].

The PdG adduct can lead to the formation of other highly genotoxic structures, including DNA-strand cross-links and DNA-protein cross-links, which may be responsible for the carcinogenesis process [53]. Furthermore, acetaldehyde can bind to proteins directly, causing structural and functional alterations; among these proteins, there is glutathione, a protein involved in the reduction of alcohol-induced oxidative stress, and enzymes involved in DNA repair and methylation. Acetaldehyde can directly inhibit the activity of DNA methyltransferase (DNMT), or it can also reduce DNMT mRNA levels, thereby reducing DNMT production [54]. Furthermore, acetaldehyde and ethanol can inhibit the synthesis of S-adenosyl-L-methionine (SAMe), essential for DNA methylation.

Under normal conditions, acetaldehyde is converted to acetate by acetaldehyde dehydrogenase (ALDH) enzymes. The main enzyme responsible for most of the conversion of acetaldehyde to acetate in the liver is the enzyme ALDH2. The ALDH2*2 allelic variant is a common polymorphism of this enzyme (especially among the East Asian populations [55]) that is responsible for lower ALDH2 activity. Carriers of this polymorphism show a slower acetaldehyde metabolism and, consequently, an accumulation of acetaldehyde that has a greater possibility of exerting the described genotoxic effects.

### 3.3. Alteration of Retinoic Acid Metabolism

Retinoids play an important function in the regulation of carcinogenesis as they can induce cell growth, cell differentiation, and apoptosis [56]. Retinoic acid primarily acts as a tumor suppressor [57]. Alcohol can interfere with retinoid metabolism by inhibiting the oxidation of vitamin A to retinoic acid [58]. Furthermore, alcohol enhances the activity of CYP2E1, which is involved in the process of metabolizing retinoic acid with the consequent production of toxic metabolites [59].

It has been reported that there is an association between chronic alcohol consumption and reduced retinoid levels in the liver [60]. A link has also been shown between low levels of retinol in the blood and an increased risk of developing head and neck cancers [61].

### 3.4. Enhancement of Inflammation

Inflammation plays a key role in cancer progression and is increased by alcohol consumption [62]. Chronic alcohol consumption can result in the recruitment of monocytes and macrophages into the tumor microenvironment, resulting in the production of pro-inflammatory cytokines, such as tumor necrosis factor (TNF-α) and the interleukins IL-1, IL-6, and IL-8 [63]. These proinflammatory cytokines determine the activation of enzymes that generate oxidants leading to the formation of ROS [64].

These cytokines also activate NF-kB, which stimulates ROS-producing enzymes. Furthermore, IL-8 is hypothesized to contribute to the accumulation of neutrophils in the liver, leading to acute inflammation [65]. Elevated levels of IL-8 have been found in patients with alcoholic hepatitis [66]. In addition, the cytokine IL-6 is involved in the production of the anti-apoptotic protein Mcl-1, with the reduction in the apoptotic mechanism and greater exposure of the cell to DNA damage [67].

### 3.5. DNA Methylation

Chronic alcohol consumption can alter the epigenetic profile of the individual by destroying the intracellular reserves of methyl groups that support the epigenome and 1CM [68]. Methylation of the 5′-cytosine-phosphate-guanine-3′ (CpG) island is an important epigenetic mechanism that controls gene activity [69]. Following alcohol consumption, the DNA methylation status is altered such that some oncogenes are upregulated and tumor suppressor genes are downregulated [6].

DNMTs are de novo methyltransferases; polymorphisms in the DNMT promoter region can increase promoter activity, leading to hypermethylation of genes, including tumor suppressor genes [70]. Alcohol can “neutralize” or reduce the hypermethylating activity of DNMT genetic variants [71].

Hypomethylation and gene expression can be affected by alcohol at the level of endogenous antioxidants [72]. For example, alcohol can cause a reduction in glutathione levels, acting on the trans-sulfuration pathway, and thus increases ROS-induced stress, which ultimately further shifts from SAM production [73].

## 4. Mechanisms of Alcohol Action in the Pathogenesis of Breast Cancer

**Figure 2:**
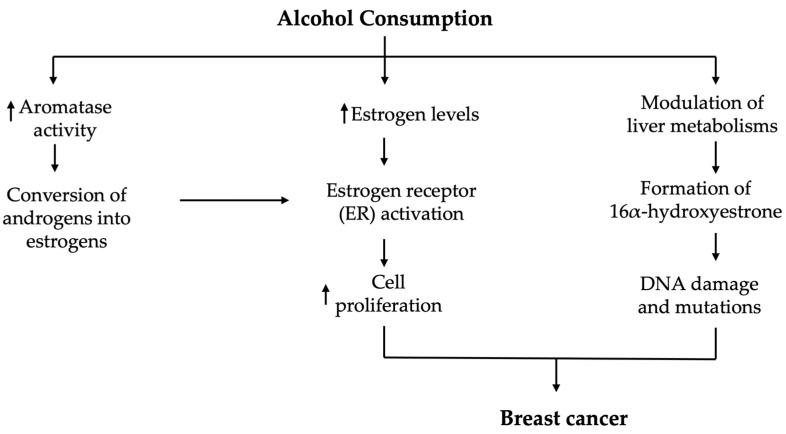
Alcohol consumption can increase the risk of breast cancer by affecting estrogen metabolism. Chronic alcohol intake increases circulating estrogen levels, promoting the growth of estrogen receptor (ER)-positive breast cancer cells. Alcohol also enhances the activity of aromatase, resulting in the conversion of androgens into estrogens, especially in peripheral tissues like adipose tissue. This results in higher local concentrations of estrogen in breast tissue, which further stimulates estrogen receptor signaling and cell proliferation. Additionally, alcohol can alter the metabolism of estrogen in the liver, leading to the production of 16α-hydroxyestrone, which can cause DNA damage and mutations, contributing to the development of breast cancer. The short pointing up/down arrows indicate increase/decrease.

The association between alcohol consumption and increased breast cancer risk is well-established in the literature, but the exact mechanisms underlying this relationship remain complex and multifactorial [74]. Several biological pathways have been proposed to explain how alcohol contributes to the initiation and progression of breast cancer (Figure 2). These mechanisms include alterations in hormonal regulation, the formation of carcinogenic metabolites such as acetaldehyde, the induction of oxidative stress, and the promotion of chronic inflammation [1].

An association has been observed between alcohol abuse and advanced and invasive breast cancer [75], so alcohol might promote the progression of existing tumors and induce more aggressive phenotypes. Many recent experimental studies support this hypothesis and show that alcohol consumption increases the aggressiveness and malignancy of breast cancer [76,77].

### 4.1. Hormonal Regulation and Estrogenic Effects

One of the primary mechanisms by which alcohol consumption is believed to increase breast cancer risk is through its effects on hormone metabolism, particularly estrogen. One of the key unique mechanisms in alcohol-induced breast cancer is its influence on estrogen metabolism [78]. Estrogens play a key role in the etiology of breast cancer [40]. It has been observed that chronic alcohol consumption is associated with an alteration in estrogen levels and an increase in the expression and activity of the estrogen receptor (ER-α) [79]. Alcohol has been shown to influence both the synthesis and metabolism of estrogen [80]. Alcohol consumption, especially at higher levels, has been consistently associated with elevated levels of circulating estrogens, which are known to promote the growth of estrogen receptor (ER)-positive breast cancer cells [81]. Estrogen acts as a growth factor for breast tissue by binding to estrogen receptors on the surface of cells, activating signaling pathways that promote cell proliferation.

In women, the majority of breast cancers express estrogen receptors, making them responsive to this hormonal signaling [82]. Moreover, alcohol has been shown to enhance the activity of aromatase, the enzyme responsible for the conversion of androgens (e.g., testosterone) into estrogens in peripheral tissues, including adipose tissue [83]. This increased aromatase activity leads to higher local concentrations of estrogen in breast tissue, further promoting estrogen receptor signaling and cellular proliferation. Alcohol may also affect the metabolism of estrogen, leading to an increased production of estrogen metabolites that are more carcinogenic [84]. In both premenopausal and postmenopausal women, alcohol increases E2 levels by promoting aromatase activity, thereby raising estrogenic stimulation in estrogen receptor-positive (ER+) breast cancer cells. In postmenopausal women, alcohol can also increase the conversion of androstenedione (an androgen) to E1 in peripheral tissues, particularly in adipose tissue, further contributing to elevated estrogen levels [85,86].

The liver, which is the primary organ responsible for estrogen metabolism, converts estradiol into various metabolites, some of which are more likely to form DNA adducts or induce oxidative damage in cells [87]. Alcohol can alter this metabolic pathway by modulating liver enzyme activity, potentially increasing the formation of 16α-hydroxyestrone, a metabolite thought to be more carcinogenic compared to other metabolites like 2-hydroxyestrone. This shift in estrogen metabolism may increase breast cancer risk by promoting DNA damage and mutations [88].

In addition to estrogen receptor-positive breast cancer, alcohol consumption has also been associated with an increased risk of HER2-positive (HER2+) breast cancer [89]. For HER2+ breast cancer, alcohol may contribute to tumor progression by influencing growth factors and signaling pathways. Alcohol-induced inflammation and oxidative stress can activate signaling molecules like NF-kB, which may promote HER2 receptor overexpression, a key feature of HER2+ cancers. In the case of triple-negative breast cancer, which lacks estrogen, progesterone, and HER2 receptors, alcohol’s role in promoting carcinogenesis is less understood but still significant [90,91].

### 4.2. Acetaldehyde and DNA Damage

Another significant mechanism through which alcohol contributes to breast cancer risk involves the formation of acetaldehyde, the primary metabolite of ethanol. Acetaldehyde is a known carcinogen that has been shown to directly damage DNA and interfere with DNA repair mechanisms, promoting mutations and genomic instability, both of which are key events in carcinogenesis [92].

Chronic alcohol consumption can cause DNA damage and genetic mutations [93]. Although alcohol itself is not a direct carcinogen, its metabolism produces acetaldehyde, which can form adducts with proteins and DNA, inducing genetic mutations, DNA cross-linking, and chromosomal aberrations [1,3,94]. The expression of ADH, a key enzyme of alcohol metabolism, has been demonstrated in human breast epithelial cells; therefore, human breast tissue can metabolize alcohol [95]. Due to the reduced ability of breast tissue to detoxify acetaldehyde, it accumulates for prolonged periods, increasing its toxicity [96]. It is therefore hypothesized that alcohol exposure induces breast carcinogenesis through the production of acetaldehyde [77].

### 4.3. Oxidative Stress and Reactive Oxygen Species

Oxidative stress, caused by an imbalance between ROS production and the body’s ability to neutralize them with antioxidants, is another critical mechanism linking alcohol consumption with breast cancer. Alcohol metabolism generates ROS, which can directly damage cellular structures, including DNA, lipids, and proteins [97].

The liver metabolizes ethanol primarily via two enzymes: alcohol dehydrogenase and aldehyde dehydrogenase. The intermediate product, acetaldehyde, is further metabolized to acetate by ALDH [98]. ROS are produced because of the incomplete reduction of oxygen during the enzymatic reactions, including the conversion of ethanol to acetaldehyde by ADH and the subsequent conversion of acetaldehyde to acetate by ALDH. During this process, ROS are generated as by-products, which can accumulate and cause oxidative damage. ROS can induce mutations in the mitochondrial and nuclear DNA, damage cell membranes, and promote inflammatory processes, all of which contribute to carcinogenesis [99]. ROS can directly damage mitochondrial DNA (mtDNA), leading to mutations in mtDNA. Furthermore, ROS can damage cell membranes by oxidizing lipids (lipid peroxidation), leading to membrane fluidity changes, leakage of cellular contents, and ultimately cell death. Thus, alcohol-induced ROS production and mitochondrial dysfunction play a significant role in the initiation and progression of carcinogenesis.

Chronic alcohol consumption has been shown to cause mitochondrial dysfunction, further exacerbating oxidative stress [100]. Mitochondria are a key source of ROS, and their dysfunction can result in an enhanced production of these reactive molecules. Mitochondrial DNA (mtDNA) is particularly vulnerable to oxidative damage, as it lacks the same protective mechanisms as nuclear DNA, such as histone proteins and efficient DNA repair systems. Accumulated mtDNA mutations can disrupt cellular metabolism and contribute to cancer cell proliferation [101].

ROS are capable of inducing DNA strand breaks, base modifications, and cross-linking, leading to mutations in critical genes involved in cell cycle regulation (e.g., TP53), apoptosis (e.g., BAX), and DNA repair (e.g., BRCA1/2) [102]. These mutations can provide a selective advantage for cancerous cells, allowing them to proliferate uncontrollably.

### 4.4. Epigenetic Modifications

Furthermore, alcohol also contributes to breast carcinogenesis via dysregulation of epigenetic regulation of gene expression, particularly abnormal DNA methylation, as epigenetic dysregulation is a key mechanism for tumor initiation and progression [103].

Recent research has highlighted the role of epigenetic alterations in cancer development, and alcohol-induced epigenetic modifications may contribute to breast cancer risk. Alcohol can influence gene expression through changes in DNA methylation, histone modification, and microRNA expression, all of which can have profound effects on tumor suppression and oncogene activation.

Chronic alcohol consumption has been shown to alter DNA methylation, particularly by inducing genome-wide hypomethylation and tumor suppressor gene hypermethylation [104,105]. Hypermethylation of CpG island promoters of genes involved in central cellular pathways, such as DNA repair (e.g., BRCA1) and cell cycle control (e.g., p16INK4a), can lead to their silencing, thus promoting cell proliferation and carcinogenesis [54,106].

Alcohol may also affect the acetylation and methylation of histone proteins, leading to changes in chromatin structure that alter gene expression. Such modifications can activate oncogenes or silence tumor suppressor genes, contributing to breast cancer development [6,72,107].

## 5. Mechanisms of Alcohol Action in the Pathogenesis of Ovarian Cancer

While alcohol consumption is a well-established risk factor for several types of cancer, including breast cancer, its role in the pathogenesis of ovarian cancer is less understood and less studied. However, emerging evidence suggests that alcohol may influence ovarian cancer development through several biological mechanisms, including alterations in hormonal regulation, the induction of oxidative stress, inflammation, acetaldehyde-induced DNA damage, and epigenetic modifications. Ovarian tumors often exist within a tumor microenvironment rich in inflammatory cytokines, and alcohol’s role in promoting inflammation may further support tumor growth and metastasis [108]. Despite this, the exact relationship between alcohol consumption and ovarian cancer risk requires more focused research to determine the strength and nature of this association.

### 5.1. Hormonal Regulation and Estrogenic Effects

Like breast cancer, ovarian cancer is influenced by hormonal factors, and alcohol’s effects on hormone regulation are thought to play a key role in the development of this malignancy. Although the ovary is the primary organ responsible for estrogen production, alcohol can modulate the balance of sex hormones in the body, thereby influencing ovarian cancer risk [34].

Alcohol consumption has been shown to increase circulating levels of estrogens, particularly estradiol, which is the most potent form of estrogen. Elevated estrogen levels can promote the growth of hormone-dependent ovarian cancers, particularly those of the epithelial subtype, which accounts for the majority of ovarian cancer cases [109]. Estrogen acts by binding to estrogen receptors (ER-α and ER-β) on ovarian epithelial cells, activating downstream signaling pathways that regulate cell proliferation, survival, and apoptosis. In cases of excess estrogen, this signaling can become dysregulated, potentially leading to the uncontrolled growth of malignant cells [110].

In addition to estrogens, alcohol consumption may disrupt the balance of other hormones, including progesterone and androgens. Progesterone plays a protective role against ovarian cancer by counteracting the proliferative effects of estrogen. Chronic alcohol consumption can lead to lower levels of progesterone, potentially increasing the mitogenic effects of estrogen and promoting the growth of estrogen-dependent ovarian tumors [111].

### 5.2. Acetaldehyde and DNA Damage

Acetaldehyde is a potent carcinogen that can contribute to the development of various cancers, including ovarian cancer.

Acetaldehyde can bind to DNA, forming adducts that cause structural changes in the DNA molecule [92]. These DNA adducts can lead to mutations if they are not repaired by the cell’s DNA repair mechanisms. These mutations may occur in key tumor suppressor genes (such as TP53) or oncogenes (such as MYC), contributing to the initiation of ovarian cancer [112].

Acetaldehyde also interferes with DNA repair processes by inhibiting the activity of enzymes involved in DNA repair, such as poly(ADP-ribose) polymerase (PARP) [113,114]. Acetaldehyde’s inhibitory effect on DNA repair may enhance the likelihood of genetic alterations that promote ovarian cancer [115].

### 5.3. Oxidative Stress and Reactive Oxygen Species

Alcohol metabolism produces reactive oxygen species, which can induce oxidative damage in tissues throughout the body, including the ovaries. The prolonged exposure to ROS can drive the transformation of normal ovarian cells into malignant ones [116].

Ethanol consumption, particularly chronic or excessive drinking, results in increased ROS generation, leading to oxidative stress in ovarian cells [117]. ROS generated by alcohol metabolism can directly cause DNA damage by inducing base modifications, strand breaks, and cross-linking. The cumulative DNA damage resulting from ROS exposure increases the risk of mutations in oncogenes or tumor suppressor genes, such as KRAS, BRCA1, BRCA2, and TP53, all of which are frequently altered in ovarian cancer [118]. This DNA damage can lead to genetic instability, which allows cancer cells to proliferate uncontrollably.

Alcohol-induced oxidative stress can damage the mitochondria, leading to mitochondrial dysfunction [101]. Dysfunctional mitochondria contribute to further ROS production, creating a positive feedback loop that exacerbates oxidative stress and accelerates the onset of cancer [119].

## 6. Discussion and Conclusions

Alcohol consumption has been shown to elevate levels of circulating estrogens, particularly through increased aromatase activity and adipose tissue conversion of androgens to estrogens, leading to enhanced cell proliferation in hormone-sensitive tissues. In the context of ovarian cancer, while the evidence is less definitive, emerging studies suggest that alcohol may also influence risk through similar pathways.

Additionally, the induction of oxidative stress from alcohol metabolism contributes to DNA damage and inflammation, both of which are recognized precursors to cancer development. For instance, the formation of reactive oxygen species and the subsequent oxidative modifications to cellular components can create a microenvironment conducive to tumorigenesis in ovarian tissues. Furthermore, genetic polymorphisms in alcohol-metabolizing enzymes, such as ALDH2, may influence individual susceptibility to alcohol-related cancer risks. These genetic factors, combined with environmental influences, underscore the complexity of the relationship between alcohol and cancer.

These findings highlight the urgent need for further research to fully elucidate the mechanisms by which alcohol influences cancer development in breast and ovarian tissues. The epidemiological evidence firmly supports the association between alcohol abuse and an increased risk of breast cancer, with a dose–response relationship that emphasizes the risks of both moderate and heavy drinking. The mechanisms underlying this association involve hormonal changes, oxidative stress, acetaldehyde formation, and inflammation, all of which contribute to carcinogenesis in breast tissue. Although the evidence linking alcohol consumption to ovarian cancer is less conclusive, emerging studies suggest a possible modest association, warranting further investigation into the underlying biological mechanisms.

Given the public health implications of alcohol abuse, particularly concerning cancer risk, public health strategies aimed at reducing alcohol consumption could play a significant role in the prevention of both breast and ovarian cancers.

Controlling alcohol-related breast and ovarian cancers requires a multifaceted approach, incorporating pharmacological agents, lifestyle interventions, and early detection strategies. Furthermore, alcohol metabolism generates reactive oxygen species (ROSs) that induce DNA damage, contributing to genetic mutations and tumor progression. By incorporating antioxidant therapies to counteract oxidative stress, the damaging effects of ROS could be mitigated, potentially decreasing cancer risk and progression.

Reducing alcohol intake remains one of the most effective preventive measures, particularly for individuals with a family history of these cancers or known genetic predispositions. Combining these strategies with a greater understanding of the molecular mechanisms of alcohol-mediated carcinogenesis provides a promising avenue for reducing the burden of alcohol-associated breast and ovarian cancers.

## Data Availability

Not applicable.
